# Supplementary material for: Depressive Symptoms and Their Associated Factors in Vocational–Technical School Students during the COVID-19 Pandemic
Source: Int J Environ Res Public Health. 2022 Mar 21;19(6):3735. doi: 10.3390/ijerph19063735 (PMC8955985; doi:10.3390/ijerph19063735)
Supplement: Supplementary file 1 [file ijerph-19-03735-s001.zip › ijerph-1595979-supplementary.pdf]

International Journal of
Environmental Research and Public Health

MDPI

Table S1. Poisson regression analysis of depressive symptoms and independent variables (n=343).

Variables	Prevalence of Depressive Symptoms				
	(%)	PR (95% CI)	*p*	Adjusted PR (95% CI)	*p*
Sociodemographic characteristics					
Sex					
Male	31.6	1	<0.001	1	<0.001
Female	52.9	1.68 (1.28–2.19)		1.72 (1.31–2.27)	
Age (years)					
14-15	39.4	1	0.461	1	0.475
16	47.4	1.20 (0.89–1.63)		1.19 (0.88–1.48)	
17-18	42.3	1.08 (0.78–1.49)		1.08 (0.79–1.49)	
Color/ ethnicity					
White	42.9	1	0.980	1	0.901
Brown	43.6	1.02 (0.78–1.32)		1.06 (0.82–1.37)	
Other	23.5	1.04 (0.71–1.52)		1.02 (0.70–1.48)	
Area					
Rural	43.3	1	0.960	1	0.306
Metropolitan	43.6	1.01 (0.78–1.30)		0.87 (0.67–1.13)	
Grade/ Year					
11th grade/ 3rd year	18.1	1	0.194	1	0.003
10th grade/ 2nd year	36.2	1.39 (0.97–1.99)		1.80 (1.18–2.76)	
9th grade/ 1st year	45.6	1.33 (0.93–1.89)		2.12 (1.37–3.26)	
Computer					
Yes	41.7	1	0.218	1	0.127
No	49.4	1.18 (0.91–1.55)		1.16 (0.89–1.52)	
Employment					
No	42.7	1	0.616	1	0.193
Yes	45.8	1.07 (0.82–1.41)		1.20 (0.91–157)	
Household					
Lives with the father and the mother	37.6	1	0.024	1	0.109
Lives with only the mother or only with the father	52.8	1.40 (1.10–1.80)		1.29 (1.01–1.66)	
Does not live with the parents	40.7	1.09 (0.66–1.77)		1.01 (0.64–1.61)	
Pandemic-related situations					
COVID-19 diagnosis					
No	44.0	1	0.592	1	0.720
Yes	39.5	0.90 (0.61–1.33)		0.93 (0.64–1.36)	
Social isolation					
Not at all isolated	34.3	1	<0.001	1	0.034
Slightly isolated	27.9	0.82 (0.45–1.48)		0.79 (0.38–1.63)	
Moderately isolated	43.0	1.25 (0.76–2.06)		1.17 (0.63–2.19)	
Very isolated	50.7	1.48 (0.89–2.47)		1.40 (0.73–2.72)	
Extremely isolated	75.9	2.21 (1.34–3.66)		2.04 (1.00–4.14)	
Concerned with the health of family/ friends					
Not at all concerned/Slightly	44.2	1	0.324	1	0.542
Moderately concerned	35.2	0.80 (0.51–1.24)		0.75 (0.43–1.34)	
Very concerned/Extremely	45.9	1.04 (0.74–1.45)		0.95 (0.60–1.50)	
Concerned with remote learning					
Not at all concerned/Slightly	45.8	1	0.246	1	0.521
Moderately concerned	31.3	0.68 (0.37–1.25)		0.67 (0.31–1.46)	
Very concerned/Extremely	45.4	0.99 (0.63–1.56)		0.89 (0.48–1.67)	
Concerned with the impact of COVID-19 on finances					
Not at all concerned/Slightly	39.5	1	0.094	1	0.382
Moderately concerned	36.3	0.92 (0.63–1.34)		0.90 (0.55–1.45)	
Very concerned/Extremely	49.4	1.25 (0.92–1.69)		1.18 (0.78–1.78)	
Social distancing measures					
I followed all social distancing measures	53.2	1	0.165	1	0.235

I left home to perform essential activities, including work	46.2	0.87 (0.64–1.89)		0.85 (0.62–1.16)	
I left home only to perform essential activities, unrelated to work	37.6	0.71 (0.52–0.96)		0.73 (0.54–0.99)	
I did not follow any social distancing measure	44.4	0.84 (0.47–1.47)		0.93 (0.51–1.70)	
Social distancing period					
Fewer than 30 days	42.4	1		1	
From 30 to 60 days	40.4	0.95 (0.65–1.40)	0.733	0.94 (0.64–1.38)	0.951
More than 60 days	45.3	1.07 (0.78–1.50)		0.97 (0.62–1.52)	
Eating habits					
Feeling hungry in the last 7 days					
Never	37.9	1		1	
Rarely	67.5	1.78 (1.37–2.31)	<0.001	1.82 (1.43–2.32)	<0.001
Sometimes/ often/ always	77.8	2.05 (1.54–2.74)		1.78 (1.33–2.39)	
Eating fruits in the last 7 days					
Regular consumption[a]	37.1	1	0.039	1	0.067
Irregular consumption	48.4	1.31 (1.01–1.68)		1.26 (0.98–1.62)	
Eating legumes/ vegetables in the last 7 days					
Regular consumption[a]	42.0	1	0.485	1	0.431
Irregular consumption	45.8	1.09 (0.85–1.39)		1.10 (0.87–1.40)	
Drinking soda in the last 7 days					
Irregular consumption	44.4	1	0.564	1	0.646
Regular consumption[a]	41.0	0.92 (0.70–1.21)		1.07 (0.81–1.40)	
Psychoactive substance use					
Tobacco smoking					
No	42.4	1	0.256	1	0.440
Yes	51.2	1.21 (0.87–1.68)		1.14 (0.82–1.59)	
Smoked in the last 30 days					
Never	43.1	1	0.405	1	0.339
1 or more days	55.6	1.29 (0.71–2.34)		1.32 (0.75–2.38)	
Alcohol drinking					
No	40.4	1	0.198	1	0.310
Yes	47.3	1.17 (0.92–1.49)		1.14 (0.89–1.46)	
Drank in the last 30 days					
Never	40.4	1		1	
1-2 days	48.6	1.21 (0.84–1.73)	0.006	1.12 (0.79–1.58)	0.121
3-9 days	69.6	1.72 (1.27–2.34)		1.45 (1.06–1.99)	
10 or more days	50.0	1.24 (0.61–2.51)		1.39 (0.76–2.53)	
Being drunk					
Never	41.4	1		1	
1-2 days	45.9	1.11 (0.76–1.62)	0.072	0.97 (0.63–1.50)	0.262
3 or more days	60.7	1.47 (1.05–2.04)		1.33 (0.90–1.98)	
Prior drug use (marijuana, cocaine, crack)					
No	42.6	1	0.036	1	0.081
Yes	66.7	1.57 (1.03–2.38)		1.44 (0.96–2.17)	
Drug use in the last 30 days					
Never	43.2	1	0.294		
1 or more days	66.7	1.54 (0.69–3.46)		1.28 (0.48–3.40)	0.623
Sexual behavior					
Prior sexual intercourse					
No	40.6	1	0.057	1	0.068
Yes	51.7	1.28 (0.99–1.64)		1.28 (0.98–1.69)	
Age at first sexual intercourse					
≥15 years	52.6	1		1	
13 - 14 years	44.0	0.84 (0.50–1.39)	0.328	0.88 (0.52–1.52)	0.413
≤12 years	71.4	1.36 (0.80–2.30)		1.38 (0.75–2.53)	
Number of sexual partners in lifetime					
1	57.5	1		1	
2 to 3	41.9	0.73 (0.45–1.19)	0.441	0.74 (0.45–1.19)	0.389
4 or more	55.6	0.97 (0.59–1.58)		1.08 (0.64–1.85)	
Physical activity level					

High	28.3	1		1	
Moderate	42.8	1.51 (0.97–2.34)	0.014	1.41 (0.92–2.16)	0.047
Low	52.7	1.86 (1.20–2.89)		1.68 (1.09–2.59)	
WHOQOL-bref					
High QoL	13.2	1		1	
Moderate QoL	40.0	3.04 (1.80–5.12)	<0.001	2.87 (1.68–4.89)	<0.001
Low QoL	77.2	5.87 (3.62–9.50)		5.66 (3.48–9.19)	

Acronyms: CI, confidence interval; PR, prevalence ratio; WHOQOL-bref, World Health Organization; QoL, Quality of Life.

p values in bold indicate significant differences ($p < 0.05$).
